# Competition and Drought Alter Optimal Stomatal Strategy in Tree Seedlings

**DOI:** 10.3389/fpls.2020.00478

**Published:** 2020-05-08

**Authors:** Nicole Zenes, Kelly L. Kerr, Anna T. Trugman, William R. L. Anderegg

**Affiliations:** ^1^School of Biological Sciences, University of Utah, Salt Lake City, UT, United States; ^2^Department of Geography, University of California, Santa Barbara, Santa Barbara, CA, United States

**Keywords:** plant hydraulics, drought, competition, stomatal optimization, water stress

## Abstract

A better understanding of plant stomatal strategies holds strong promise for improving predictions of vegetation responses to drought because stomata are the primary mechanism through which plants mitigate water stress. It has been assumed that plants regulate stomata to maintain a constant marginal water use efficiency and forego carbon gain when water is scarce. However, recent hypotheses pose that plants maximize carbon assimilation while also accounting for the risk of hydraulic damage via cavitation and hydraulic failure. This “gain-risk” framework incorporates competition in stomatal regulation because it takes into account that neighboring plants can “steal” unused water. This study utilizes stomatal models representing both the water use efficiency and carbon-maximization frameworks, and empirical data from three species in a potted growth chamber experiment, to investigate the effects of drought and competition on seedling stomatal strategy. We found that drought and competition responses in the empirical data were best explained by the carbon-maximization hypothesis and that both drought and competition affected stomatal strategy. Interestingly, stomatal responses differed substantially by species, with seedlings employing a riskier strategy when planted with a high water use competitor, and seedlings employing a more conservative strategy when planted with a low water use competitor. Lower water users in general had less stomatal sensitivity to decreasing Ψ*_L_* compared to moderate to high water users. Repeated water stress also resulted in legacy effects on plant stomatal behavior, increasing stomatal sensitivity (i.e., conservative behavior) even when the seedling was returned to well-watered conditions. These results indicate that stomatal strategies are dynamic and change with climate and competition stressors. Therefore, incorporating mechanisms that allow for stomatal behavioral changes in response to water limitation may be an important step to improving carbon cycle projections in coupled climate-Earth system models.

## Introduction

Stomata are small pores on plant leaves that control the rate at which carbon is gained and water is lost. Stomata are the primary mechanism through which plants can mitigate water stress (Jones and Sutherland, 1991) and often respond first to changes in environmental conditions and hormone signaling (Schroeder et al., 2001) by opening or closing on short timescales (i.e., minutes) to regulate stomatal conductance and gas exchange rates. Many studies have investigated the sensitivity of stomatal conductance to environmental drivers including soil moisture (Ali et al., 1998) and humidity (Aasamaa and Sober, 2010) as well as physiological metrics that are indicative of changes in environmental stimuli including drought-induced changes in leaf abscisic acid concentrations (a stress hormone in leaves; Bahrun et al., 2002) and leaf water potential (Ψ_L_; Lawlor and Tezara, 2009). While substantial progress has been made in understanding the physiology underlying stomatal regulation, we currently lack a fully mechanistic understanding. Thus, optimal stomatal behavior theories, where stomata aim to maximize fitness, hold substantial promise for mechanistically predicting stomatal behavior (Cowan and Farquhar, 1977; Katul et al., 2009; Medlyn et al., 2011; Wolf et al., 2016). The stomatal models based on optimal behavior theory are built on the critical trade-off between carbon uptake and water loss, particularly during unfavorable environmental conditions, and can inform predictions of plant productivity and survival under potential novel future climate conditions.

The literature related to optimal stomatal behavior theories is extremely rich, and largely began with seminal work by Cowan and Farquhar (1977) which has been subsequently extended to many environmental conditions and species (Katul et al., 2009; Manzoni et al., 2011; Medlyn et al., 2011; Lin et al., 2015; Buckley, 2017). The Cowan and Farquhar theory was developed using the assumption that plants maximize photosynthesis (A_N_) over time while limiting transpiration (E). “Optimal” stomatal behavior occurs when δA_N_/δE (the marginal water use efficiency) is equal to a constant λ (or 1/λ in some formulations) (Cowan and Farquhar, 1977; Buckley, 2017). Under this water use efficiency (WUE) hypothesis, plants adjust stomatal conductance to maintain a constant δA_N_/δE ratio over a given period of time, which is often not specified but has been studied with timeframes spanning from a day to multiple seasons in the literature (Manzoni et al., 2013; Novick et al., 2016; Anderegg et al., 2018). This theory, however, does not account for competition between plants for a shared water supply, which is a critical component of terrestrial ecosystem dynamics given widespread root system overlap (Casper and Jackson, 1997).

**FIGURE 1:**
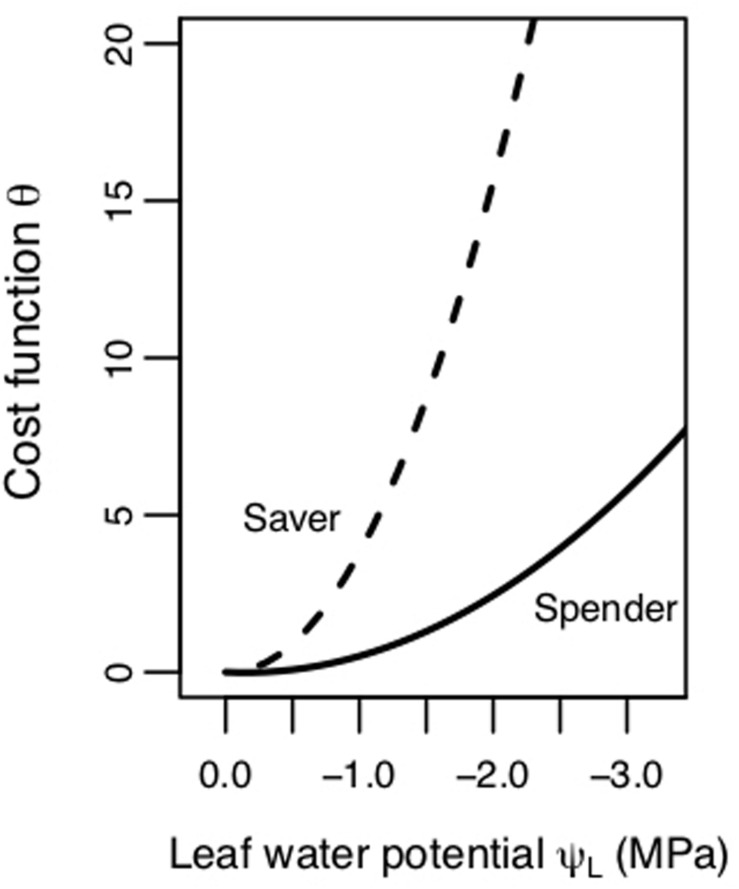
Schematic of hydraulic cost/risk function (θ, unitless) as a function of leaf water potential (Ψ_L_, MPa). One strategy is a riskier “spender” strategy (lower β_1_ or slope value, shallower cost function) with stomata closing at a more negative Ψ_L_. The other strategy is a more conservative “saver” strategy (higher β_1_ or slope value, steeper cost function) with stomata closing faster as Ψ_L_ declines.

More recent studies have proposed and employed a “gain-risk” carbon maximization (CM) hypothesis that optimizes stomatal conductance based on photosynthetic gain versus the cost or risks to the hydraulic continuum associated with decreases in Ψ_L_ (Prentice et al., 2014; Wolf et al., 2016; Sperry et al., 2017; Anderegg et al., 2018; Eller et al., 2018; Venturas et al., 2018). The carbon maximization hypothesis uses a game theory approach where plants are under selective pressure to prevent both short- and long-term consequences associated with water limitation, namely the risk of hydraulic damage via cavitation and hydraulic failure associated with low Ψ_L_, to simulate the effects of competition (Wolf et al., 2016). Under the CM hypothesis, optimal stomatal behavior aims to maximize A_N_ while minimizing a hydraulic cost/risk term [defined here as θ(Ψ_L_)], at a given Ψ_L_ and set of environmental conditions. With this model, hydraulic cost or risk to the plant increases with declining Ψ_L_. The steepness of this cost function indicates different plant physiological strategies for dealing with water stress. Plants with cost functions with steeper slopes follow a “water saver” strategy, and close stomata earlier as Ψ_L_ declines (Figure 1). Plants employing a “water spender” strategy tend to keep stomata open longer because their cost increases at a slower rate with more negative Ψ (Figure 1).

Stomatal behavior, in response to environmental and competitive cues, is modulated by a suite of physiological traits that regulate response to abiotic stress and avoid mortality while competing with neighbors for scarce resources (Piutti and Cescatti, 1997; Bottero et al., 2017). On short to moderate timescales (days to months), plant hydraulic and photosynthetic traits can plastically respond to the environment and buffer plant water stress during drought and competition (Callaway et al., 2003; Bartlett et al., 2014). These traits include the water potential at which cells lose turgor (turgor loss point, Ψ_TLP_), leaf photosynthetic rate (expressed as the maximum rate of carboxylation, V_cmax_), and hydraulic conductivity of different plant tissues (K). In addition, plants balance competitive capacity with the risk of hydraulic damage to xylem tissue, which can result in a long-term reduction in photosynthesis (Anderegg et al., 2014; Mackay et al., 2015; Trugman et al., 2018). Indeed, damage to water transport tissue is one major mechanism through which reduced photosynthetic capacity (Resco et al., 2009) or even plant death (Allen et al., 2010; Phillips et al., 2010; Anderegg et al., 2015) can occur. Thus, untangling the mechanisms underlying how plants mediate hydraulic risk in balance with carbon gain is critical for predicting tree survival and productivity.

Moving forward, critical questions remain about the efficacy of different optimization hypotheses (CM vs. WUE) and whether stomatal strategy is an inherent and constrained trait with little plasticity or whether plants behavior changes with environmental conditions and competitive environment. While several studies have investigated optimized stomatal behavior in response to drought alone (Sperry and Love, 2015; Anderegg et al., 2018; Lu et al., 2019), our understanding of this behavior in response to the complex interactions between drought and competition is limited. Therefore, we ask: (1) Do stomatal responses to environmental variation support the WUE or CM stomatal theory; (2) Does competition affect the sensitivity of stomatal closure to Ψ_L_ (i.e., cost function steepness); (3) Do plants change their stomatal behavior following drought; and (4) Is stomatal behavior explained by concomitant changes in other hydraulic metrics? Critically, answers to these questions will significantly advance our understanding of stomatal behavior because our experiment allows us to directly examine the effects of competition on stomatal strategy.

## Materials and Methods

### Study Design

We conducted a growth chamber experiment in which *Populus tremuloides*, *Populus angustifolia*, and *Pinus ponderosa* (referred to hereafter as aspen, cottonwood, and pine, respectively) seedlings were planted with a competitor or alone and subsequently subject to multiple periods of water stress. We describe the design and measurements briefly here, full details are in Kerr et al. (unpublished). We chose these species because they co-exist in natural stands where competition is likely to occur, and they employ a spectrum of water use strategies ranging from high/profligate water users (cottonwoods), to intermediate (aspen), and low/conservative (pine) (Anderegg and HilleRisLambers, 2016). Each seedling was either grown alone in an 18-l square pot with 15 l of soil or in competition with another seedling in a 36-l rectangular (i.e., two 18 L square pots connected together) pot with 30 l of soil with another seedling to maintain the same amount of relative resources (Supplementary Figure S1). There were six replicates of the following planting groups: aspen grown alone (A), cottonwood grown alone (C), pine grown alone (P), aspen competing with another aspen (AxA), aspen competing with cottonwood (AxC), and aspen competing with pine (AxP) (Supplementary Figure S2). As one of the most widespread tree species in North America, aspen is important across a vast diversity of ecosystems. However, it has been found to be sensitive to drought and susceptible to drought-induced mortality (Anderegg et al., 2012; Worrall et al., 2013). Therefore, in order to accommodate space constraints of the growth chamber, we chose aspen to be our focal species when designing the study.

The baseline conditions in the growth chamber were set to 25°C temperature, 75% relative humidity, 1150 μmol m^–2^ s^–1^ photosynthetic photon flux density, and 400 ppm ambient carbon dioxide (CO_2_). Photoperiod for the growth chamber was set to closely match that of the greenhouse, where the seedlings were initially grown, with lights on from 6:00 to 19:15 using EYE HORTILUX ceramic metal halide 315 W grow lamp lights. During the predrought treatment period, we weighed a subset of pots and calculated a baseline average water volume to be given daily. Then we imposed three water limitation treatments sequentially – a low soil moisture treatment, an elevated vapor pressure deficit (VPD) treatment, and a combination of both simultaneously – on the seedlings. We took gas exchange and hydraulic measurements during the control predrought period, each treatment period, and a subsequent a post treatment recovery period. Each drought treatment lasted 5 days and seedlings were allowed to recover for 3 days in between treatments by returning to both baseline watering and growth chamber conditions. During the soil drought, we gave seedlings 50% of the water they were receiving during the predrought baseline period. During the elevated VPD treatment, watering was returned to the predrought water regime, and relative humidity was reduced from ∼75% to ∼45%. For the combination drought, reduced watering to 25% of their daily water and reduced relative humidity to 45% to impose the most significant water stress (Supplementary Table S1). Post drought treatments, the seedlings were returned to the predrought (control) conditions for 3 days.

We then fitted the WUE and CM optimization models to observed stomatal conductance measurements and compared the best fits to determine which hypothesis more skillfully predicted observed stomatal responses. Next, we evaluated how the hydraulic risk function related to competition treatment, water stress, and plant traits. The performance of the WUE and CM models, and how the hydraulic cost/risk function varied, provides insight into species’ stomatal strategies and their dynamics. We describe the modeling approach in detail below.

### Modeling Photosynthesis, Water Transport and Stomatal Conductance

We fitted our data with a stomatal optimization model that uses well-established equations for modeling photosynthesis, hydraulic conductivity, and water transport and can use either λ (i.e., the WUE theory) or θ(Ψ_L_) (i.e., the CM theory) for the optimality criterion (Anderegg et al., 2018). The core components of the model are as follows. For photosynthesis, Farquhar et al. (1980) (eqn. 1) was used to model net carbon assimilation (A_N_) as the smallest of two limiting factors; rubisco limitation (*w*_c_) and light limitation (*w*_j_).

(1) $$A_{N}=\min\left(w_{c},w_{j}\right)-R_{d}$$

where R_d_ is the rate of dark respiration. The relationship between carbon assimilation and stomatal conductance was calculated using Equation 2, based on Fick’s Law,

(2) $$A_{N}=\frac{g_{S}\,\left(C_{a}-C_{i}\right)}{1.6}$$

where, g_s_ is stomatal conductance, C_a_ is the atmospheric concentration of CO_2_, C_i_ is the internal concentration of CO_2_ inside the leaf, and 1.6 accounts for the difference in diffusion rates of water vapor and CO_2_.

Transpiration is represented by Equation 3,

(3) $$E=g_{S}\,\left(e_{S}-e_{a}\right)$$

where e_s_ is the saturated vapor pressure inside the leaf and e_a_ is the actual vapor pressure of the air.

Steady-state transpiration is modeled by Equation 4,

(4) $$E=\int_{\psi \,L}^{\psi \,S}K\,\left(\psi \right)\,d\psi$$

where Ψ*_S_* is soil water potential, and K(Ψ) is the xylem conductance function. Conductance is calculated by Equation 5

(5) $$K\,\left(\psi_{L}\right)=K_{\max}*\exp^{\left(-{\left(\frac{P}{-B}\right)}^{C}\right)}$$

where C and B Weibull curve parameters were estimated from the stem vulnerability curves (see physiological measurements below) using a bootstrapping method (Hacke et al., 2015), P is leaf water potential, and K_max_ is the whole plant maximum xylem conductance. In our modeling framework, K_max_ was unknown, thus we estimated K_max_ assuming that plants want to maximize productivity without compromising the hydraulic system. Thus, calculated to maximize the difference between measured predawn and calculated midday Ψ*_L_* while not exceeding the median water potential where 50% of maximum hydraulic conductivity is lost (P_50_), and satisfy the remaining equations (Eqn. 1–5) according to the methods in Anderegg et al. (2018). For the aspen grown alone and cottonwood grown with a competitor planting groups, in order to provide solutions for the remaining equations with viable priors for β_1_ and λ, the estimated K_max_ resulted in a midday Ψ*_L_* that exceeded P50 with Ψ*_L_* = −3.00 MPa (P50 = −2.69 MPa) and Ψ*_L_* = −1.35 MPa (P50 = −1.31 MPa), respectively.

### Modeling Plant Response to Water Stress

To understand how plants balance carbon gain versus risk/cost during water stress, we used an optimality equation that relies on measurements of stomatal conductance to estimate a “shadow cost” (or risk to future plant performance) function for both the WUE and CM theory. As described in Wolf et al. (2016) the marginal xylem tension efficiency (MXTE) is the amount of carbon gain a plant is willing to forgo to prevent a decrease in Ψ*_L_*. Importantly, the MXTE differs between the WUE and CM optimality theories as shown by Equations 6 and 7:

(6) $$M\,X\,T\,E_{W\,U\,E}=\lambda \,\frac{\delta \,E}{\delta \,\psi_{L}}$$

and

(7) $$M\,X\,T\,E_{C\,M}=\frac{\delta \,\theta }{\delta \,\psi_{L}}$$

where λ is the constant marginal water use efficiency, E is transpiration, θ is the cost/risk term.

We linearized the derivative of the cost function such that

(8) $$\frac{\delta \,\theta }{\delta \,\psi_{L}}=\beta_{1}\,\psi_{L}+\beta_{0}$$

where β_1_ and β_0_ are parameters fitted using observational stomatal conductance measurements. The linearized form of the derivative is advantageous in that it (i) distinguishes between the increasing versus decreasing responses of δθ/δΨ to decreases in Ψ*_L_* in the CM and WUE hypotheses, respectively, and (ii) minimizes unconstrained parameters. This linear marginal cost function implies a parabolic form of the cost/risk function with declining water potential (Anderegg et al., 2018):

(9) $$\theta \,\left(\psi_{L}\right)=\frac{\beta_{1}}{2}\,\psi_{L}^{2}+\beta_{0}\,\psi_{L}+c$$

where c is the intercept of the cost function, which is not solved for because the derivative gives the necessary information to quantify stomatal strategies.

### Parameter Estimation

A Markov-Chain Monte Carlo (MCMC) algorithm was used to calculate the posterior probability density function (PDF) of β_1_ or λ that provided the best fit between observed and modeled stomatal conductance for the CM or WUE models, respectively. MCMCs were run for each of the WUE and CM models for species type (aspen, cottonwood, and pine) and each planting group (competition and no competition), for a total of eight different fitting groups per model. For the CM model, we found that the best β_1_ and β_0_ often covaried, leading to an equifinality issue (eq. 8). Therefore, we ran a series of MCMCs (∼50) each using a different fixed value of β_0_ in order to estimate β_1_. For each of the eight fitting groups, we determined the range of initial β_0_ values (Table 1) that would allow a solution to the system of equations with variables in biologically realistic bounds (Ψ*_L_* within [−10,0]) for each planting group using an initial value of β_1_ = 0.1. This value was chosen because a positive β_1_ represents a marginal increase in cost of damage with decreasing Ψ*_L_*, so it is physiologically realistic, but is still a relatively uninformed initial guess. When running the series of MCMCs, our range and increment of β_0_ values (Table 1) was chosen because it allowed us to explore the full parameter space while maintaining a level of reasonable computational efficiency. Each separate MCMC was run for 5000 steps for each of the fixed β_0_. The first 1000 steps were discarded as burn in and we sampled for every tenth step to account for temporal autocorrelation to represent the posterior PDF. The mode of the posterior PDF was used as the estimated β_1_.

**TABLE 1:** Range of fixed β_0_ values used to estimate β_1_.

Planting group	Lowest β_0_	Highest β_0_	Sequenced by
Aspen alone (A)	−7.9	0.6	0.1
Aspen × aspen (AxA)	−10.9	0.6	0.1
Aspen × cottonwood (AxC)	−9	0.6	0.1
Aspen × pine (AxP)	−12.4	0.4	0.2
Cottonwood alone (C)	−8.1	0.3	0.2
Cottonwood × aspen (CxA)	−15.7	0.5	0.2
Pine alone (P)	−4.6	0.5	0.1
Pine × aspen (PxA)	−4.1	0.4	0.1

*Values allow the MCMC to be run with a prior start value of β_1_ = 0.1. Three planting groups; aspen competing with pines (AxP), cottonwoods grown alone (C), and cottonwoods competing with aspen (CxA), were sequenced by 0.2 due to computing power available.*

For the WUE model, for each of the eight planting groups, we ran MCMCs with an initial guess of λ = 0.1. For some fitting groups, no solution to the system of equations could be found at small λ values so we gradually increased our initial guess by increments of 0.1 until a solution could be obtained. When proposing the next step running the MCMCs, we confine lambda to the positive parameter space as a negative value would imply a decrease in cost as Ψ_L_ becomes more negative. We used linear models and the Akaike Information Criterion (AIC) score for all three species to assess model performance and determined that the CM model more skillfully predicted stomatal conductance compared to the WUE model for all three of our species. Thus, for subsequent analyses we used only the CM model.

When investigating effects of competition between planting groups, we performed one fit using all the stomatal conductance measurements for each planting group taken across the five treatment periods for the CM only. When looking at effects of treatment period within a planting group, we performed separate fits using the subset of stomatal conductance measurements taken at each treatment period; predrought, soil drought, VPD drought, combination soil and VPD drought, and post drought recovery. Because we only calculated V_cmax_ during the predrought and post drought recovery periods, we used the predrought measured V_cmax_ values for the predrought and three drought periods and the post drought measured V_cmax_ for the post drought recovery treatment period (Kerr et al., unpublished).

### Physiological Measurements

At each treatment period, we took gas exchange measurements using a Li-6400 open gas exchange system with a red-blue light source and conditions set to maintain 25°C leaf temperature, 1200 μmol m^–2^ s^–1^ photosynthetic photon flux density, 400 ppm ambient CO_2_, and relative humidity matching that of the growth chamber for the current treatment period. Predawn leaf water potential (Ψ*_PD_*) was measured for each seedling using a Scholander-type pressure chamber before the growth chamber lights turned on (between the hours of 0400 and 0600) (Figures 3D–F). Samples for Ψ*_PD_* were removed from the plant, placed in a sealed plastic bag, and water potential was measured within 5 min.

In the predrought and post drought periods, we measured vulnerability curves using the centrifuge method (Alder et al., 1997) and calculated the water potential at which 50 percent of the xylem conductivity is lost (P50). Using the standard flow method, we calculated percent loss of conductivity (PLC) comparing the native stem conductivity to the maximum hydraulic conductivity. V_cmax_ was determined from constructing A-Ci curves using a Li-6400 open gas exchange system and settings of 25°C leaf temperature, 1200 μmol m^–2^ s^–1^ photosynthetic photon flux density, 400 ppm ambient CO_2_, and relative humidity matching that of the growth chamber. We also calculated the Ψ_TLP_ in the predrought and recovery periods using the pressure-volume method (Tyree and Hammel, 1972) with a Scholander-type pressure chamber and mass balance to measure Ψ*_L_* and weight as samples periodically dried.

### Analyses of Results

To answer our first question, we used the best fit parameters as determined from our initial MCMC simulations using the eight planting groups for each the WUE and CM models. Separate MCMC fits were calculated for each planting group and we used linear models and the Akaike Information Criterion (AIC) score to assess relative model performance.

To answer our second and third questions, we performed non-parametric analyses of longitudinal time series data for comparisons of β_1_ values over time (treatment periods), comparisons of β_1_ values between planting groups, and the interaction between time and planting group using R package (nparLD) (Noguchi et al., 2012) with β_0_ representing repeated measures subjects in an ANOVA analysis. ANOVA-type statistics are reported, and Tukey HSD *post hoc* pairwise comparisons were conducted. For hydraulic metrics, we performed linear regressions for mean predrought and post drought recovery β_1_ and TLP, PLC, and Ψ*_PD_* for each species.

## Results

**FIGURE 2:**
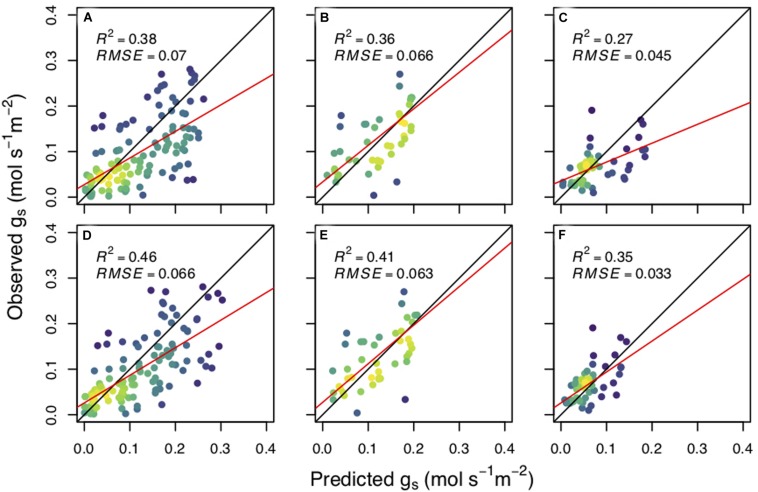
The predicted stomatal conductance (g_s_) versus observed stomatal conductance (mol s^– 1^ m^– 2^). Upper panel values were calculated using the water use efficiency model for each species; *Populus tremuloides* **(A)**, *Populus angustifolia* **(B)**, and *Pinus ponderosa* **(C)**. The bottom panels were calculated using the carbon maximization model for each species (**D–F**, respectively). β_0_ and best fit β_1_ were estimated for each planting group and plotted together for predictive power per species. Black lines represent the 1:1 line, red lines are the best fit for linear regression and adjusted *R*^2^ and root mean square error (RMSE) values are reported. Yellow indicates a higher density of points while purple represents a lower density.

We found that the CM model more skillfully predicted stomatal conductance compared to the WUE model for all three of our species: aspen (*R*^2^_WUE_ = 0.38, *R*^2^_CM_ = 0.46) (Figures 2A,D), cottonwood (*R*^2^_WUE_ = 0.42, *R*^2^_CM_ = 0.54) (Figures 2B,E), and pine (*R*^2^_WUE_ = 0.27, *R*^2^_CM_ = 0.35) (Figures 2C,F). The CM model also had a lower AIC than the WUE model for all species: aspen_WUE_ = −331.9, aspen_CM_ = −349.1; cottonwood_WUE_ = −218.5, cottonwood_CM_ = −235.2; pine_WUE_ = −201.6, pine_CM_ = −207.6.

**FIGURE 3:**
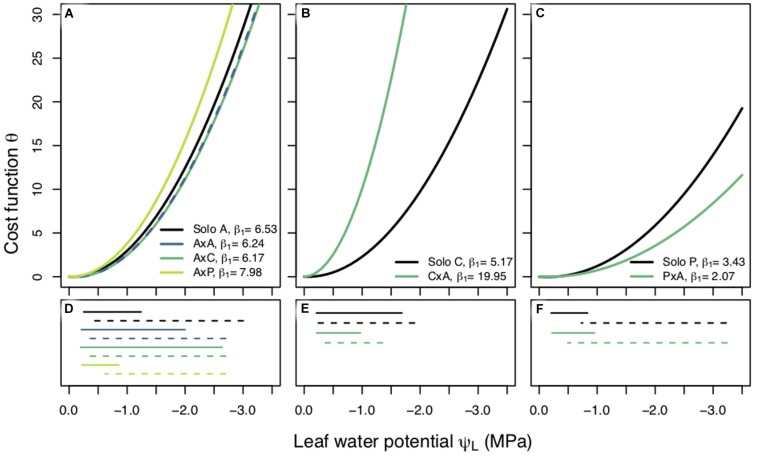
Cost functions (θ) between planting groups within a species plotted against leaf water potential (Ψ_L_, MPa). Each curve is produced using the best fit β_1_ value and the corresponding fixed β_0_. Best fit β_1_ values are reported for each planting group. Panels are separated by species; *Populus tremuloides* **(A)**, *Populus angustifolia* **(B)**, and *Pinus ponderosa* **(C)** and each planting group is represented by a different color. Measured predawn (Ψ_PD_, solid lines), and modeled midday (Ψ_MD_, dashed lines), water potentials plotted under their corresponding species **(D–F)**. Legend key for planting groups: aspen grown alone (A), aspen competing with aspen (AxA), aspen competing with cottonwoods (AxC), aspen competing with pines (AxP); cottonwoods grown alone (C), cottonwoods competing with aspen (CxA); pines grown alone (P), and pines competing with aspen (PxA).

We found different responses to competition across our three species. The results of a non-parametric two-way repeated measures ANOVA for aspen showed that planting group had a significant effect on β_1_ and the *post hoc* Tukey HSD pairwise test indicated that competitor identity, in addition to competitor presence, had an effect on β_1_. Specifically, a high water use competitor, such as aspen and cottonwood, resulted in a riskier stomatal strategy than when aspen were grown alone or with a pine (Figure 3A). Cottonwoods grown with a competitor saw a shift to a larger β_1_ value and cost function with a steeper slope (Figure 3B) (*p* < 0.0001) indicating cottonwood seedlings’ stomatal behavior was more conservative under competition. Interestingly, pines had the opposite response: pines under competition employed a riskier stomatal strategy, had a small β_1_ value (*p* < 0.0001), and a cost function with a shallower slope (Figure 3C). β_1_ values also varied across species: cottonwoods had largest β_1_, aspens had a more moderate β_1_, and pines had the smallest β_1_, indicating that water use strategy may relate to the rate of stomatal closure as Ψ*_L_* declines.

**FIGURE 4:**
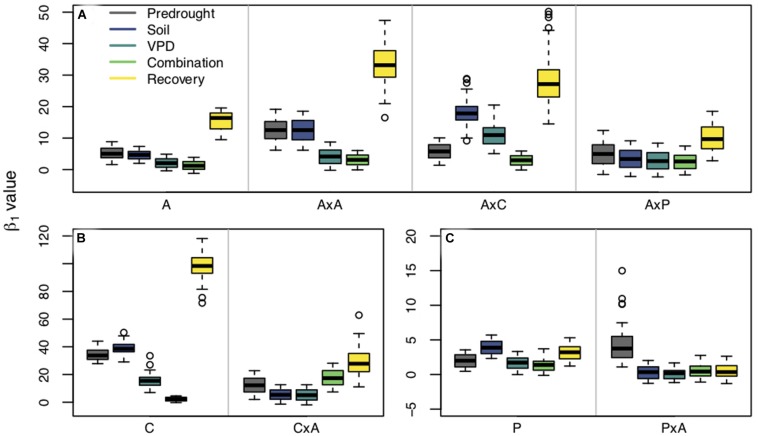
β_1_ values estimated from fixed β_0_ values within planting groups across treatment periods [predrought, soil drought, vapor pressure deficit (VPD) drought, combination soil and VPD drought, and post drought recovery]. Panels are separated by species: *Populus tremuloides* **(A)**, *Populus angustifolia* **(B)**, and *Pinus ponderosa* **(C)**. Planting groups are separated within panels by gray lines: aspen grown alone (A), aspen competing with aspen (AxA), aspen competing with cottonwoods (AxC), aspen competing with pines (AxP); cottonwoods grown alone (C), cottonwoods competing with aspen (CxA); pines grown alone (P), and pine competing with aspen (PxA). Colors represent treatment periods. Boxplots show the median, 25th and 75th percentiles with whiskers extending to furthest data point up to 1.5x the interquartile range, and data points beyond this are considered outliers and represented by open circles. *Y*-axes vary between species.

Planting group, treatment, and their interaction all had an effect on β_1_ (Figure 4). Repeated measures tests were significant for differences between planting groups, treatment period, and the interaction between group and period (*p* < 0.0001) for all three species. For aspen and cottonwoods, all treatment periods pairwise comparisons were significant (*p* < 0.01) indicating that both drought presence and type resulted in shifts in β_1_. Pines saw less of an effect of treatment periods on β_1_, likely due to the fact that pines experienced less severe water stress compared to aspens and cottonwoods (as verified through Ψ*_PD_*). However, pine recovery β_1_ were significantly different from predrought β_1_, VPD drought β_1_, and combination drought β_1_ (*p* < 0.0001), indicating that there was an effect of water stress on pine stomatal behavior.

Interactions between planting group and treatment period had a variety of responses (Table 2). Aspen grown alone, aspen competing with cottonwoods (AxC), and aspen competing with pine (AxP) had no significant differences in β_1_ values during the predrought period, but all four planting groups β_1_ values were significantly different in the post-drought recovery period, indicating that there was an effect of competitor identity on changes in β_1_ following drought treatments (*p* < 0.0001). Within each of the aspen planting groups, recovery β_1_ values were significantly different from all other time periods providing further evidence that drought affected stomatal behavior (*p* < 0.0001). High water use competitors, such as the case when aspen competed with aspen (AxA) or cottonwood (AxC), resulted in larger recovery β_1_, while the aspen grown alone and aspen competing with pine resulted in a smaller recovery β_1_ (Figure 4A), suggesting increased competition for water stimulates a more dramatic shift toward a more conservative stomatal strategy post water stress treatment (after drought).

**TABLE 2:** *Post hoc* Tukey HSD pairwise interaction comparisons for *Populus tremuloides, Populus angustifolia*, and *Pinus ponderosa*.

(a) Planting group	Treatment period	Pairwise comparisons within planting groups			
A	Predrought	A			
	Soil	A			
	VPD		B		
	Combination		B		
	Recovery			C	
AxA	Predrought	A			
	Soil	A			
	VPD		B		
	Combination		B		
	Recovery			C	
AxC	Predrought	A			
	Soil		B		
	VPD			C	
	Combination				D
	Recovery					E
AxP	Predrought	A			
	Soil	A	B		
	VPD	A	B		
	Combination		B		
	Recovery			C	
C	Predrought	A			
	Soil		B		
	VPD			C	
	Combination				D
	Recovery					E
CxA	Predrought	A			
	Soil		B		
	VPD		B		
	Combination			C	
	Recovery				D
P	Predrought	A			
	Soil		B		
	VPD	A			
	Combination	A			
	Recovery		B		
PxA	Predrought	A			
	Soil		B		
	VPD		B		
	Combination		B		
	Recovery		B		

**(b) Treatment period**	**Aspen planting groups**	**Pairwise comparisons within treatment periods**		**Cottonwood planting groups**	**Pairwise comparisons within treatment periods**		**Pine planting groups**	**Pairwise comparisons within treatment periods**	

Predrought	A	a		C	a		P	a	
	AxA		b	CxA		b	PxA		b
	AxC	a							
	AxP	a							
Soil	A	a		C	a		P	a	
	AxA		b	CxA		b	PxA		b
	AxC		c						
	AxP	a	b						
VPD	A	a		C	a		P	a	
	AxA		b	CxA		b	PxA		b
									

**(b)Treatment period**	**Aspen planting groups**	**Pairwise comparisons within treatment periods**		**Cottonwood planting groups**	**Pairwise comparisons within treatment periods**		**Pine planting groups**	**Pairwise comparisons within treatment periods**	

	AxC		c						
	AxP	a	b						
Combination	A	a		C	a		P	a	
	AxA		b	CxA		b	PxA		b
	AxC	a	b						
	AxP	a	b						
Recovery	A	a		C	a		P	a	
	AxA	b		CxA		b	PxA		b
	AxC		c						
	AxP		d						

*Letters represent statistically significant differences (*p* < 0.05). Panel (a) shows within planting group comparisons across treatment periods. Panel (b) shows within treatment period and between planting group comparisons. Planting groups are aspen grown alone (A), aspen competing with aspen (AxA), aspen competing with cottonwoods (AxC), aspen competing with pines (AxP), cottonwoods grown alone (C), cottonwood competing with aspen (CxA), pines grown alone (P) and pines competing with aspen (PxA).*

Within cottonwood planting groups, the majority of treatment periods were significantly different (*p* < 0.05) and their recovery β_1_ values were largest, indicating that repeated drought treatments led to a shift toward a more conservative stomatal strategy. Competitors also affected how seedlings responded to each sequential drought: solo cottonwoods and competing cottonwoods had different β_1_ values during all treatment periods (Figure 4B). Pines grown without a competitor responded to the drought treatments and shifted to a more conservative stomatal, as indicated by a larger recovery β_1_ compared to pretreatment β_1_, VPD treatment β_1_, and combination drought treatment β_1_ (*p* < 0.001). Contrary to patterns seen in the other species and planting groups, pine competing with aspen (PxA) saw a statistically significant larger β_1_ in the predrought period than the drought and post drought treatment periods. Within treatment periods, pines without a competitor and pines with a competitor were always significantly different (*p* < 0.05), indicating an effect of planting group on stomatal strategy (Figure 4C).

**FIGURE 5:**
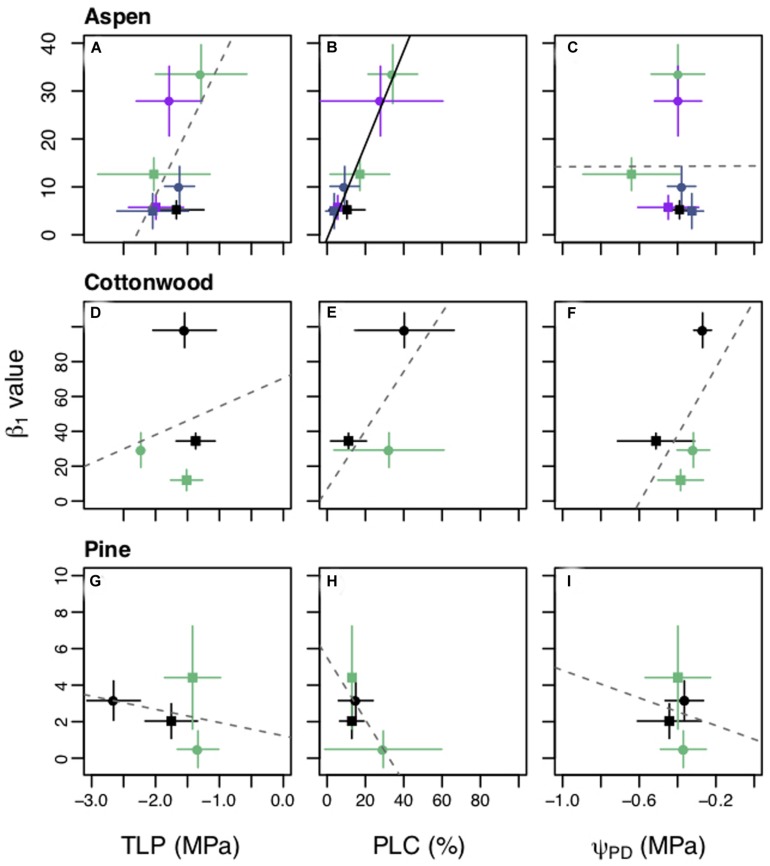
Comparison of β_1_ values with turgor loss point (TLP) **(A,D,G)**, percent loss of hydraulic conductivity (PLC) **(D,E,H)**, and predawn water potential (Ψ_PD_) **(C,F,I)** during the predrought and post drought recovery treatment periods for each planting group. Panels are organized by species: *Populus tremuloides* **(A–C)**, *Populus angustifolia* **(D–F)**, *Pinus ponderosa* **(G–I)**. Squares (■) are predrought measurements and circles (•) are post drought measurements. Each planting group is represented by color, groups for the plants grown alone are in black. Aspen competing with aspen (AxA), cottonwood competing with aspen (CxA), and pine competing with aspen (PxA) groups are in green; aspen competing with cottonwoods (AxC) is in purple; and aspen competing with pine (AxP) is in blue. Mean values are plotted with ± one standard deviation bars shown. Solid black lines represent significant results (*p* < 0.05) and gray dashed lines for non-significant results for the best fit for ordinary least squares regression. *Y*-axes vary by species.

We found there were relationships between changes in physiological metrics and changes in β_1_ such that a decrease in drought resistance and increase in hydraulic damage lead to a more conservative strategy in aspen and cottonwoods (Figures 5A–F). Larger β_1_ values were correlated with less negative TLP and increased PLC for aspens (Figures 5A,B). However, only the PLC relationship was statistically significant (*p* < 0.01). Larger β_1_ values were correlated with less negative TLP, PLC, and less negative Ψ*_PD_* for cottonwoods (Figures 5D–F), although none were statistically significant. In contrast to the aspens and cottonwoods, pines saw a non-significant negative correlation with all three measurements (Figures 5G–I), such that stomatal closure rate decreased in step with increased PLC and less negative TLP.

## Discussion

Here we show the CM model more skillfully predicted stomatal response to water stress induced through both competition and drought compared to the WUE model. Further, we found that both competition and drought influenced stomatal strategies. Critically, the effects of competition were complicated and varied by species. Surprisingly, pines exhibited a riskier, “spenders” strategy in response to competition whereas cottonwoods exhibited a more conservative, “savers” strategy, which is counterintuitive based on our current understanding of their life history strategies. However, the competitor’s water use strategy helps explain the magnitude and direction of shift in cost function with seedlings adopting a riskier strategy when competing with higher water users and a more conservative strategy when competing with lower water users. All three species showed this pattern across treatment periods with varying environmental conditions and watering regimes, and the magnitude of these shifts was likely related to the strength of competition., These results illustrate that gas exchange variation in individual trees and whole forest communities is likely influenced by a complex interplay among environmental stress, competitive stress, and stomatal and trait strategies.

Shifts in the shadow cost (MXTE) and pricing hydraulic risk in response to both competition and drought were explained by competitor species and adjustments in physiological traits. The angiosperm species had a positive correlation between PLC and β_1_ such that the seedlings had both a higher PLC and larger β_1_ during treatment recovery compared to the pretreatment period. The coordination between PLC and β_1_ could be due to an increase in embolism, which would limit the amount of water seedlings could transport, causing a shift toward a “savers” stomatal behavior to avoid additional hydraulic damage (Figure 1). The relationships between larger β_1_ and shifts in, TLP, PLC, and Ψ*_PD_* were consistent with our hypotheses that increased hydraulic damage (i.e., higher PLC) and lower drought tolerance (less negative TLP) are associated with a shift toward a more conservative stomatal strategy. In addition, cottonwoods had less negative Ψ*_PD_* in the recovery period, which is indicative of lower water stress, but also larger β_1_, further supporting evidence of hydraulic damage and increased cost even at less negative Ψ*_L_*. The pines, the only gymnosperm, had non-significant but slightly negative correlations between β_1_ and TLP, PLC, and Ψ*_PD_* and showed less variation in these variables, both within planting groups and across treatment periods (Figures 5H,I). This may be due to the fact that the pines were not significantly stressed during the drought treatments, resulting in less of a response in both β_1_ and hydraulic metrics.

Increased stomatal sensitivity following drought events could have important impacts on productivity, even under well-water conditions, preventing plants from photosynthesizing and repairing hydraulic damage sustained during drought (Brodersen and McElrone, 2013). This physiological response could help to explain a lag in growth recovery and mortality that has been observed following drought events (Anderegg et al., 2015; Peltier et al., 2016; Klockow et al., 2018; Trugman et al., 2018). This hypothesis is supported by other studies: Aasamaa and Sober (2011) found trees exposed to drought showed an increase in stomatal sensitivity to changes in environmental conditions following a recovery period. A positive correlation between PLC and β_1_ would make it more difficult for trees to prepare for future drought events as they are forgoing carbon even under low atmospheric demand, due to increased stomatal sensitivity. Therefore, avoiding damage to hydraulic tissue and the increased carbon cost of recovering hydraulic conductivity could favor the selection for plants to become more conservative following drought periods (Brodribb et al., 2010). Some of these shifts in stomatal strategy could be due to changes in biochemical mechanisms, such as abscisic acid (ABA) concentrations, that have been shown to respond to drought and influence optimal stomatal behavior (Haworth et al., 2018; Brunetti et al., 2019). For example, increased foliar ABA has been shown to maintain stomatal closure when plants were returned to well-watered conditions (Tombesi et al., 2015). Investigating how long plants take to revert to their predrought strategy, as well as the mechanisms driving the adjustment, could give insight into ecosystem dynamics shifts following changes in frequency and severity of droughts.

While there have been a number of studies addressing the role of physiological traits and mechanisms affecting plant response during drought (Farooq et al., 2009), the recovery of photosynthetic rates, Ψ*_PD_*, and leaf gas exchange in plant communities after natural drought has not been as thoroughly investigated (Flexas et al., 2006). The shifts we observed toward a more conservative stomatal strategy and more rapid stomatal closure as Ψ*_L_* declined may help to explain the lag in gas exchange recovery following drought events, even when Ψ*_L_* return to predrought levels (Pšidová et al., 2015). Indeed, Yin and Bauerle (2017) found that incomplete post-drought recovery was present across all plant functional types documented in their meta-analysis, although the magnitude varied greatly. Damage to hydraulic transport tissue has been found to be a major determinant of photosynthetic recovery in the desert perennial tree *Prosopis velutina* due to the increase in stomatal limitation even without changes to leaf biochemistry (Resco et al., 2009), which may explain the shift to a more conservative strategy with increased PLC following drought. Incorporating mechanisms that reflect underlying processes driving the changes in stomatal strategy and stomatal sensitivity into mechanistic models may help better predict changes in plant productivity following drought.

Here, we provide evidence that the CM hypothesis accurately predicts plant stomatal strategies in complex environmental and competitive stress scenarios. Further, overall stomatal behavior and shifts in stomatal strategy in response to drought were species-specific. Interestingly, higher water users showed increased sensitivity to changes in Ψ*_L_* and had a larger shift to conservative strategies after drought had ended. Crucially, we show that drought and water stress, even on short term timescales, can have lasting effects on plant stomatal behavior even when the plants returned to favorable environmental conditions. As current climate models assume perfect plant recovery from water stress, there is a need to better describe and incorporate these stomatal behavior changes and plant recovery in order to better predict ecosystem fluxes and forest response to a changing climate.

## Data Availability Statement

The datasets generated for this study are available on request to the corresponding author.

## Author Contributions

All authors designed the experiment and carried out the data interpretation. WA developed the model. KK and NZ were involved in data collection. NZ performed the analyses. NZ wrote the manuscript with contributions from all other authors.

## Conflict of Interest

The authors declare that the research was conducted in the absence of any commercial or financial relationships that could be construed as a potential conflict of interest.
