# Supplementary material for: Competition and Drought Alter Optimal Stomatal Strategy in Tree Seedlings
Source: Front Plant Sci. 2020 May 8;11:478. doi: 10.3389/fpls.2020.00478 (PMC7227391; doi:10.3389/fpls.2020.00478)
Supplement: Supplementary file 1 [file Data_Sheet_1.docx]

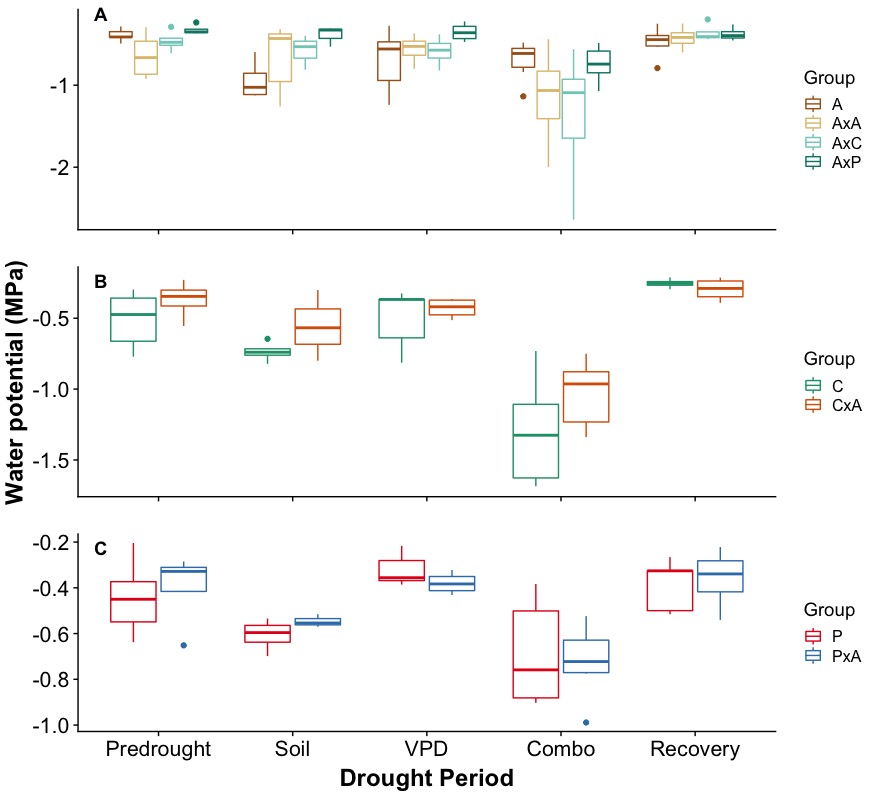
Supporting Information

**Table S1.** Mean growth chamber environmental conditions during daylight hours (06:00 to 19:15) for the predrought control, soil drought, elevated vapor pressure deficit (VPD) drought, combination soil and elevated VPD drought, and post drought recovery time periods, including temperature (°C), relative humidity (RH), and photosynthetically active radiation (PAR).

|  | Predrought | Soil drought | VPD drought | Combination drought | Recovery |
| --- | --- | --- | --- | --- | --- |
| Temperature | 25 | 25 | 27 | 25 | 25 |
| RH | 75 | 73 | 55 | 41 | 75 |
| PAR | 1105 | 1140 | 1120 | 1195 | 1200 |

**Figure S1.** Photographs of the pots and seedlings used in the study. **A**) A representative image of an aspen and a pine seedling together in a competition pot right after transplanting (Photo taken November 13, 2017). Note that both seedlings started out the study with equivalent heights and sizes. **B**) Individual and competitions pots in the greenhouse right after transplanting (Photo taken on November 13, 2017). **C**) The LiCor 6400 in use on seedlings in the growth chamber during the predrought control period (photo taken on January 8, 2018). *Note:* Seedlings had grown in size, and bamboo poles were used to help support the cottonwood seedlings as they grew taller.

**Figure S2**. Schematic of the Percival PR-915 growth chamber and experiment layout. The walk-in growth chamber was 2.4 meters wide by 4 meters long by 3 meters high (not shown in 2D schematic) and was able to hold six replicates of pots and planting groups as indicated by the numbers 1-6. Each replicate maintained the same layout of planting groups, which included individual pots of aspen (A), cottonwood (C) and pine (P) and competition pots of aspen and aspen (AxA), aspen and cottonwood (AxC), and aspen and pine (AxP). Shaded pots were randomly selected for destructive measurements during the predrought control period and were replaced by pots from the greenhouse. Replacement pots were given two weeks to acclimate to the growth chamber before implementation of the water limitation treatments.
